# Statin Intensity and Clinical Outcome in Patients with Stable Coronary Artery Disease and Very Low LDL-Cholesterol

**DOI:** 10.1371/journal.pone.0166246

**Published:** 2016-11-08

**Authors:** Soo Youn Lee, Seung-Jin Oh, Eung Ju Kim, Chi-Yoon Oum, Sung Hwan Park, Jaewon Oh, Jung-Sun Kim, Byeong-Keuk Kim, Sungha Park, Hyuk-Jae Chang, Geu-Ru Hong, Young-Guk Ko, Seok-Min Kang, Donghoon Choi, Jong-Won Ha, Myeong-Ki Hong, Yangsoo Jang, Namsik Chung, Sang-Hak Lee

**Affiliations:** 1 Division of Cardiology, Department of Internal Medicine, Severance Hospital, Yonsei University College of Medicine, Seoul, Korea; 2 Cardiovascular Research Institute, Yonsei University College of Medicine, Seoul, Korea; 3 Division of Cardiology, Department of Internal Medicine, NHIS Ilsan Hospital, Goyang, Korea; 4 Department of Cardiology, Cardiovascular Center, Korea University Guro Hospital, Seoul, Korea; 5 Department of Biostatistics and Computing, The Graduate School, Yonsei University, Seoul, Korea; University of Insubria, ITALY

## Abstract

**Background:**

Although intensive statin therapy is recommended for high risk patients, evidence of its benefit in patients with stable coronary artery disease (CAD) and very low low-density lipoprotein-cholesterol (LDL-C) has been very rare. In this study, we investigated whether higher statin intensity reduces cardiovascular risks in this population.

**Methods:**

In this retrospective study, a total of 5234 patients with stable CAD were screened at three tertiary hospitals in Korea; 449 patients (mean age: 65 years, male: 69%) with LDL-C <80 mg/dL were finally analyzed. The statin intensities were classified according to the 2013 American College of Cardiology/American Heart Association guidelines. Patients who received statins equivalent to or weaker than atorvastatin 10 mg (group 1) were compared with those who took statins equivalent to or stronger than atorvastatin 20 mg (group 2). The impact of statin intensity on major adverse cardiac events (MACE) was evaluated during follow-up.

**Results:**

Group 1 and group 2 consisted of 181 patients (40.3%) and 268 patients (59.7%), respectively. The mean LDL-C level decreased to 52 and 57 mg/dL in group 1 and group 2, respectively, during follow-up. In a median follow-up of 4.5 years, patients of group 2 had a lower incidence of MACE (30 [16.6%] vs. 12 [4.5%], p <0.001), which were mostly related to a lower incidence of coronary revascularization. Cox proportional hazard analyses identified the statin intensity of group 2 (adjusted hazard ratio: 0.25, confidence interval: 0.11–0.55, p <0.001) and the baseline high-density lipoprotein-cholesterol level as independent determinants of MACE.

**Conclusion:**

This study provides evidence that higher intensity statins are beneficial for cardiovascular outcomes in patients with stable CAD and very low LDL-C. Statins equivalent to or stronger than atorvastatin 20 mg are more effective than lower intensity statins.

## Introduction

Most recent guidelines on lipid management emphasize the aggressive lowering of low-density lipoprotein-cholesterol (LDL-C) levels in high-risk groups [1,2]. In particular, early pharmacologic therapy has been recommended for very high-risk patients, such as those with acute coronary syndrome [3]. The rationale of this strategy is based on previous studies in which the extent of the clinical benefit derived from statin therapy correlated with the level of patient risk [4]. The benefits of statins have been proven in high-risk patients, irrespective of baseline LDL-C levels [5]. For instance, statins reduced cardiovascular events even in patients with mean LDL-C levels of 116 mg/dL [6]. For high-risk patients, more intensive lipid reduction resulted in a more favorable clinical outcome [5]. This has been demonstrated repeatedly, especially in patients with acute coronary syndrome [7–9].

On the other hand, although intensive statin therapy is also recommended for patients with stable coronary artery disease (CAD), evidence of its benefits in this population is less common. In particular, data on the effects of statins in patients with stable CAD and very low baseline LDL-C levels are very rare. In the Treating to New Targets (TNT) study, a greater clinical benefit was observed with intensive statin therapy in patients with stable CAD. However, the required LDL-C level for inclusion in the study was <130 mg/dL, and no further analysis was conducted in patients with very low LDL-C level [10].

Statins are also widely used in East Asian countries. However, sufficient data have not been published on the clinical benefits of statins in Asians. In addition, an Asian background is one of the predisposing factors for statin-related adverse reactions [1]. Although physicians are concerned about the safety of statins when prescribing high intensities of these drugs to Asians, the optimal statin dose for this population, with different cardiovascular risks, is uncertain.

The aims of this study were to investigate 1) whether the statin intensity affects cardiovascular risk in Korean patients with stable CAD and very low LDL-C (<80 mg/dL) and if so, 2) which intensity can better reduce the risk.

## Methods

### Study population

**Fig 1:**
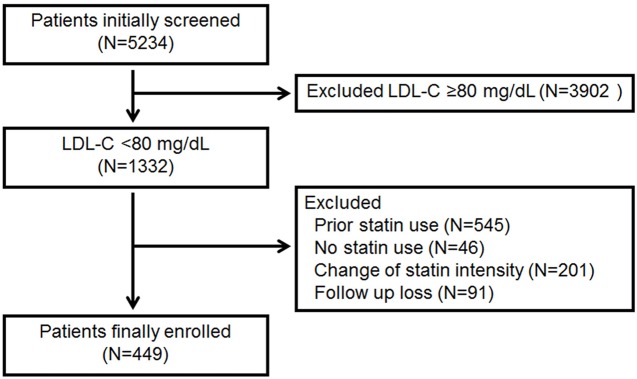
Patient screening, exclusion, and enrollment.

The Institutional Review Board (IRB) of Severance Hospital, Seoul, Korea approved this study. Informed consent was waived by the IRB for the following reasons: 1) the research involves no more than minimal risk to the subjects; 2) the waiver does not reversely affect the rights and welfare of the subjects; and 3) the research could not be practicably carried out without the waiver. Three hospitals in Korea participated in this study: Severance Hospital, Seoul; National Health Insurance Service Ilsan Hospital, Goyang; and Korea University Guro Hospital, Seoul. A total of 5234 consecutive patients who visited each hospital from May 2005 to February 2014 and had a diagnosis of stable CAD were initially screened. The diagnosis of stable CAD was made if patients had chest discomfort or pain typical of myocardial ischemia and coronary stenosis. When their symptom was not typical, diagnosis was made by further examination, including treadmill exercise test or nuclear perfusion scan. The angiographic criterion was stenosis >50% in more than one coronary artery. We excluded individuals with a history of myocardial infarction. Patients with LDL-C ≥80 mg/dL, who were taking lipid-lowering agents at the time of blood testing, or who did not take statins after the diagnosis of CAD were excluded. In addition, subjects who did not exhibit consistent statin intensity for at least 80% of the follow-up period were also excluded. Consistent statin intensity is defined as the maintenance of the same statin intensity during the study period. Finally, 449 patients were included in the analysis (Fig 1).

### Study protocol

This was a retrospective observational study. Clinical data, including demographic variables, medical history, and medication use, were obtained by trained interviewers. Blood samples were collected after a 12-h fast and analyzed by the local laboratories. The study population was treated by using standard medical therapies, including statins, according to the physicians’ discretion. The subjects were classified according to the statin intensity received during ≥80% of the follow-up period. The statin intensity was classified according to the 2013 American College of Cardiology (ACC)/American Heart Association (AHA) guidelines.

Most of our study population were prescribed with moderate intensity statins, and no patients received low intensity statins. To determine whether there was a difference in the clinical benefits experienced by patients receiving different doses of moderate intensity statins, we divided these patients into two groups: patients with atorvastatin 10 mg or similar (group 1) and those with atorvastatin 20 mg or similar. Because the numbers of patients who took high intensity statins were too small to be analyzed separately, they were combined with those received atorvastatin 20 mg or similar (group 2). We listed the frequency, dose, and treatment duration of statins in each group in S1 Table.

The patients were followed every 3–6 months in the outpatient clinic at each center. They underwent lipid profile examination every 3–12 months. Changes in LDL-C levels were calculated as follows: (final value−baseline value) / baseline value x100 (%). Clinical outcome data were obtained in outpatient clinics, by medical record reviews, or by means of telephone contact. The outcome variable was major adverse cardiac events (MACE), defined as the composite of cardiovascular death, nonfatal myocardial infarction, and coronary revascularization. A death was considered of cardiovascular origin if the cause was associated with myocardial infarcion or ischemia, arrhythmia, heart failure, or stroke, or if the death was sudden and unexpected.

### Statistical analysis

Continuous variables with little skewing are reported as means ± standard deviations. Those that do not have a normal distribution are reported as medians (interquartile ranges). The triglyceride levels did not show normal distribution. In the Kolmogorov-Smirnov tests, the p values for the baseline and follow-up triglycerides of group 1 were 0.16 and 0.12, respectively, whereas those of group 2 were 0.20 and 0.22, respectively. Therefore, we used log-transformed values for triglycerides in our analysis. Categorical variables are presented as frequencies and percentages. The patients’ clinical and laboratory variables were compared by using Student’s t-test and the chi-square test. A paired t-test was used to compare parameters before and after drug treatment within each group. As the triglyceride levels did not show a normal distribution, both the Wilcoxon signed-rank test for the median and the paired t-test for log triglycerides were used. Cumulative survival curves for each group were made by using the Kaplan-Meier method and compared by using the log-rank test. The determinants of MACE were identified through a univariate Cox regression analysis. Age, sex, and variables with p value <0.15 in the analysis were entered into a multivariate analysis in a stepwise manner. The number of events was small with regard to the number of variables entered into the multivariate analysis, and this might lead to model over-fitting. To reduce this limitation in the analysis, we performed propensity score matching. Age, sex, hypertension, diabetes mellitus, smoking, LDL-C level, and coronary artery bypass graft (CABG) were used as matching variables. Hazard ratios and 95% confidence intervals were calculated. All analyses used two-tailed tests with a significance level of 0.05. SPSS version 17.0 (SPSS Inc, Chicago, IL, USA) was used for the analyses.

## Results

### Clinical characteristics

Among 449 patients, group 1 included 181 patients (40.3%) who received atorvastatin 10 mg or similar. Group 2 included 217 patients (48.3%) who received atorvastatin 20 mg or similar and 51 patients (10.3%) who received atorvastatin 40–80 mg or similar. No patient was prescribed with low intensity statins. Patients’ ages, sexes, and medical histories did not differ between groups 1 and 2. The proportions of patients prescribed with each concomitant medication were similar among the groups. Of the total subjects, 410 (91.3%) underwent percutaneous coronary intervention, while 39 (8.7%) underwent CABG. The rates of each revascularization did not differ between the statin intensity groups (Table 1).

**Table 1:** Baseline characteristics of the patients.

	Group 1 (n = 181)	Group 2 (n = 268)	p
Age, years	65±10	65±11	0.83
Male	121 (66.9)	189 (70.5)	0.47
Medical history			
Hypertension	131 (72.4)	189 (70.5)	0.75
Diabetes mellitus	66 (36.5)	118 (44.0)	0.12
Current smoker	38 (21.0)	45 (16.8)	0.27
Body mass index, kg/m^2^	24.7±3.8	24.6±3.9	0.65
Number of diseased vessels			
1	115 (50.7)	120 (44.8)	0.42
2	59 (26.0)	80 (29.9)	
3	53 (23.3)	69 (25.4)	
Medications			
Antiplatelet agents	181 (100)	268 (100)	1.00
β-blockers	109 (60.2)	147 (54.9)	0.29
Calcium channel blockers	77 (42.5)	97 (36.2)	0.20
ACE inhibitors or ARBs	89 (49.2)	126 (47.0)	0.70
Revascularization			
PCI	170 (93.9)	240 (89.6)	0.13
CABG	11 (6.1)	28 (10.4)	
Median follow up, years (IQR)	4.9 (2.3, 6.5)	4.2 (2.3, 6.0)	0.09

Values are presented as mean ± SD, or n (%) unless otherwise stated; ACE: angiotensin converting enzyme;

ARB: angiotensin receptor blocker; PCI: percutaneous coronary intervention; CABG: coronary artery bypass graft; IQR: interquartile range

### Change in lipid profiles and clinical outcomes

The patients were followed up for a median of 4.5 years. At baseline, the mean LDL-C values were similar between the groups (65 mg/dL in group 1 and 2). Conversely, the mean follow-up LDL-C levels ranged from 52 to 57 mg/dL and were significantly lower in group 2 (p<0.001). Likewise, the percentages changes in LDL-C level were greater in group 2 (p = 0.02). At follow-up, group 2 demonstrated lower total cholesterol and similar triglyceride levels compared with those of group 2 (Table 2).

**Table 2:** Changes in lipid profiles.

		Group 1 (n = 181)	Group 2 (n = 264)	P^a^
Total cholesterol	Before	151±37	152±45	0.83
	After	129±37	119±25	0.003
	p^b^	<0.001	<0.001	
Triglycerides	Before	117 (73, 192)	104 (76,150)	0.25
	After	97 (74, 144)	102 (72, 127)	0.25
	p^b^	<0.001	0.001	
LogTriglycerides	Before	4.80±0.65	4.71±0.57	0.22
	After	4.72±0.58	4.62±0.50	0.79
	p^b^	<0.001	0.002	
HDL-C	Before	42.0±10.7	42.1±10.7	0.94
	After	44.6±12.6	42.9±10.1	0.15
	p^b^	0.001	0.34	
LDL-C	Before	65±13	65±12	0.76
	After	57±18	52±15	0.004
	p^b^	<0.001	<0.001	
% change of LDL-C		-7.7±41.9	-16.1±31.0	0.02

Values are presented as mean±SD except triglycerides, which is presented as median (IQR)

^a^: comparision between groups

^b^: comparison within a group before and after treatment; HDL-C: high-density lipoprotein-cholesterol; LDL-C: low-density lipoprotein-cholesterol

**Fig 2:**
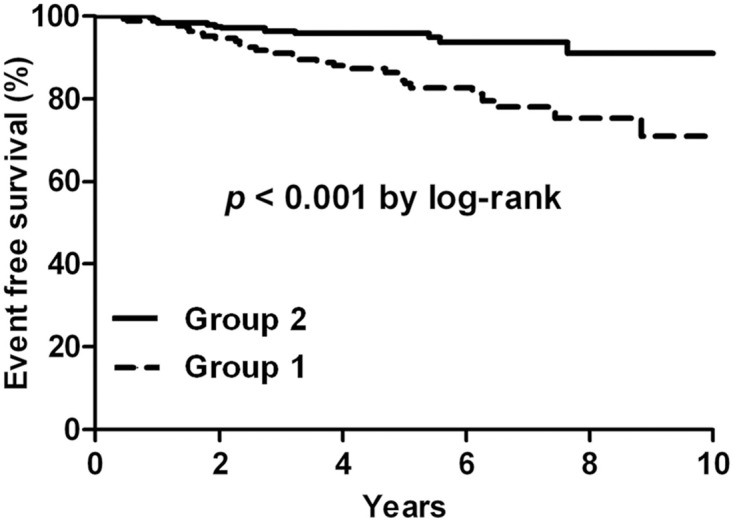
Kaplan-Meier curves for patients classified by statin intensity.

During follow-up, 42 patients (9.4%) experienced MACE, including 4 cardiovascular deaths, 2 nonfatal myocardial infarction, and 36 coronary revascularizations. Patients in group 2 had a significantly lower incidence of these events (30 [16.6%] vs 12 [4.5%], p <0.001), which were mostly associated with a lower incidence of coronary revascularization (27 [14.9%] vs 9 [3.4%], p <0.001) (Table 3). The Kaplan-Meier survival curves also revealed a higher event-free survival rate in this group (p<0.001; Fig 2).

**Table 3:** Incidence of MACE.

	Group 1 (n = 181)	Group 2 (n = 264)	p
MACE,	30 (16.6)	12 (4.5)	<0.001
Cardiovascular death	3 (1.7)	1 (0.4)	0.31
Nonfatal MI	0 (0)	2 (0.7)	0.52
Coronary revascularization	27 (14.9)	9 (3.4)	<0.001

Values are presented as n (%); MACE: major adverse cardiac events; MI: myocardial infarction

### Determinants of MACE

The results of univariate and multivariate Cox proportional hazard analyses of MACE are presented in Table 4. Older age was related to MACE, whereas a higher baseline high-density lipoprotein-cholesterol level was associated with a lower risk of MACE. With respect to statin intensity, group 2 (statins equivalent to or stronger than atorvastatin 20 mg) was associated with a lower risk of MACE, compared with group 1 (statins equivalent to atorvastatin 10 mg). After adjusting for variables derived from univariate analyses, the statin intensity of group 2 (adjusted HR: 0.25, p<0.001) and the baseline high-density lipoprotein-cholesterol (adjusted HR: 0.96, p = 0.04) were found to be independent determinants of MACE.

**Table 4:** Determinants of MACE according to Cox proportional hazard analysis.

	Unadjusted HR (95% CI)	p	Adjusted HR (95% CI)	p
Age	1.04 (1.00–1.07)	0.03	1.03 (0.99–1.07)	0.11
Male	0.92 (0.48–1.78)	0.80	--	--
Hypertension	1.01 (0.51–1.98)	0.98	--	--
Diabetes mellitus	0.87 (0.47–1.63)	0.67	--	--
Current smoker	0.92 (0.43–2.00)	0.84	--	--
Log Triglycerides	1.12 (0.64–1.97)	0.68	--	--
HDL-C	0.96 (0.93–0.99)	0.02	0.96 (0.93–0.99)	0.04
LDL-C	0.99 (0.97–1.01)	0.35	--	--
CABG	1.37 (0.54–3.50)	0.51	--	--
Statin intensity				
Group 1	Reference	--	Reference	--
Group 2	0.31 (0.16–0.61)	0.001	0.25 (0.11–0.55)	0.001

MACE: major adverse cardiac events; HR: hazard ratio; CI: confidence interval; HDL-C: high-density lipoprotein-cholesterol; LDL-C: low-density lipoprotein-cholesterol; CABG: coronary artery bypass graft

## Discussion

The present study demonstrated that 1) statin intensity is an independent determinant of MACE in patients with stable CAD, even when the baseline LDL-C is <80 mg/dL, and 2) statins equivalent to or stronger than atorvastatin 20 mg are better in reducing the risk than lower intensity statins. For the first time, to our knowledge, this study showed the clinical impact of statin intensity in patients with stable CAD and very low LDL-C level. Furthermore, our results provide evidence for an appropriate statin strategy in populations for whom further LDL-C reduction has been empiric and controversial. It is worth noting that the current data were derived from an East Asian population in whom the safety of statin is of concern and data on statin doses and clinical outcomes are extremely limited.

The data of patients with stable CAD and very low baseline LDL-C level were the focus and strength of our study. Studies on the effect of statin or statin intensity in patients with similar conditions as those in our study have been very scarce. In addition, data from this specific patient group were either not analyzed, or the sample sizes of the studies were not appropriate for drawing of conclusions. For instance, the LIPS (Lescol Intervention Prevention Study) trial, which comprised patients with stable and unstable angina, showed that fluvastatin 80 mg reduced MACE in the subgroup with LDL-C levels below the median. However, the median value was 132 mg/dL, and the study did not separately analyze the data of subjects with stable CAD and low LDL-C levels [11]. In the Heart Protection Study, simvastatin 40 mg reduced both mortality and vascular events in subjects with baseline LDL-C <116 mg/dL. However, patients with stable CAD were a minority in that trial, and the statin effect was not analyzed separately in that group [6]. The TNT trial was the only study to specifically enroll patients with stable CAD and compare the effects of different statin doses. It was remarkable that this study clearly demonstrated the superiority of higher intensity statin in subjects with stable CAD [10]. Nevertheless, because the TNT trial enrolled patients with baseline LDL-C >130 mg/dL, it is difficult to extrapolate that result to individuals with very low LDL-C levels, such as those in our study. In an analysis of the CREDO-Kyoto Registry Cohort 2, the authors demonstrated that when compared with standard intensity statins, strong intensity statins were associated, but not significantly, with better clinical outcomes. Although that rare study had a similar topic as our study and was also conducted in an Asian population, that study was rather different from ours in that 1) part of the study subjects experienced myocardial infarction; 2) the mean baseline and follow-up LDL-C were 116–125 and 92–101 mg/dL, respectively; and 3) statins classified as strong intensity in that study were classified as lower moderate intensity in our current study [12]. Therefore, we can again state that our study provides data for a specific population in which prior evidence of a benefit of aggressive lipid lowering has been absent.

Group 2 had a mean follow-up LDL-C level of 52 mg/dL and showed significant beneficial effect, whereas group 1 reached 57 mg/dL. This finding bears similarity to those of the IMPROVE-IT trial (IMProved Reduction of Outcomes: Vytorin Efficacy International Trial), which recently demonstrated that intensive lowering of LDL-C by adding ezetimibe to statin therapy, resulted in improved cardiovascular outcomes [13]. Nevertheless, the participants of the study all experienced acute coronary syndrome, which was a major difference from our study. However, interestingly, the follow-up LDL-C level in the simvastatin-ezetimibe group was 53.7 mg/dL and similar to that of group 2 in our study. In this regard, the post-treatment LDL-C levels of group 2 in our study might provide evidence of a better LDL-C target in patients with stable CAD as well.

To create a more adequate model, and minimize bias and confounding factors, propensity score matching was conducted (S2 and S3 Tables). After the matching, the incidence of MACE remained lower in group 2 (30 [16.6%] vs 6 [3.3%], p<0.001; S4 Table). A log-rank test also showed better event-free survival in group 2 (p<0.001; S1 Fig). A significant impact of higher intensity statins was further shown by Cox proportional hazard analysis (HR: 0.25, p = 0.001; S5 Table). Taken together, the benefit of higher intensity statins was consistent before and after propensity score matching.

Fifty-one patients took high intensity statins. Because only one patient (2.0%) experienced MACE during follow-up, this might have influenced the results of our study. However, in an additional analysis on group 1, group 2 minus the high intensity group, and the high intensity group separately, the statin intensity of group 2 minus the high intensity group was found to be a significant determinant of the outcome (data not shown).

Our study had some potential limitations. First, as this study was performed in a retrospective manner, clinical differences could exist between the groups. In addition, this study design can make the obtained results weaker. However, we performed propensity score matching to minimize the methodological limitation (S2–S5 Tables) and found that our major findings did not change. Second, the composition of MACE in our study could be another limitation. Although the present study showed a significant impact of statin intensity on MACE, the number of events was small and most events were coronary revascularizations, that had been driven by the physician’s choice based on ischemic symptom or objective evidence. Meanwhile, other harder events were not as frequent during the follow-up. Third, one of the inclusion criteria was that patients were required to show at least 80% consistency during the follow-up. Thus, there could have been a potential selection bias for those who were more adherent to treatment or who were taking the same statin intensity. In addition, not only the benefit but also the risk induced by a drug are important in the clinical field. Unfortunately, however, collecting data on adverse events after treatment with statins was difficult in our study, because the events were not recorded well. In a prior study in the Korean population, treatment-emergent adverse events tended to be more frequent with higher-dose statins [14]. Meanwhile, most statin-related adverse events were mild and totally reversible in other Korean studies [15, 16] and the discontinuation rate of statins has been very low even in patients receiving the maximum available dose of rosuvastatin [16]. In this regard, although data on drug adverse events were not available in our study, the risk induced by the effect might not be very large. However, we cannot completely rule out the potential risk of drug adverse events in patients prescribed with higher dose statins. Fourth, our study was not based on a sufficiently large population. However, to focus on the purpose of the study, we enrolled specific patients by using strict inclusion criteria: stable CAD, very low LDL-C level, no lipid-lowering agent at baseline, and consistent statin intensity. Thus, the final sample size was 449 patients and this is the largest study among those that have analyzed this specific group of patients. On the other hand, the current data indicate that the “high” intensity statin described in 2013 ACC/AHA guidelines has not been commonly used in CAD patients with very low LDL-C level in Korea. In this regard, our study was not appropriate for evaluating the effect of high intensity statins.

Our study, which was conducted in an East Asian population, demonstrated that higher intensity statins produce beneficial cardiovascular outcomes in patients with stable CAD and LDL-C levels <80 mg/dL. Statins equivalent to or stronger than atorvastatin 20 mg were more effective than lower intensity statins.

## Supporting Information

S1 Fig Kaplan-Meier curves for patient groups with propensity score matching. (TIF)

S1 File Minimal data set. (XLSX)

S1 Table Frequency, doses, and treatment duration of statins in each group. (DOCX)

S2 Table Baseline characteristics of the patients with propensity score matching. (DOCX)

S3 Table Changes in lipid profiles with propensity score matching. (DOCX)

S4 Table Incidence of MACE with propensity score matching. (DOCX)

S5 Table Determinants of MACE according to Cox proportional hazard analysis with propensity score matching. (DOCX)
