# Supplementary figures and images for: Statin Intensity and Clinical Outcome in Patients with Stable Coronary Artery Disease and Very Low LDL-Cholesterol
Source: PLoS One. 2016 Nov 8;11(11):e0166246. doi: 10.1371/journal.pone.0166246 (PMC5100958; doi:10.1371/journal.pone.0166246)

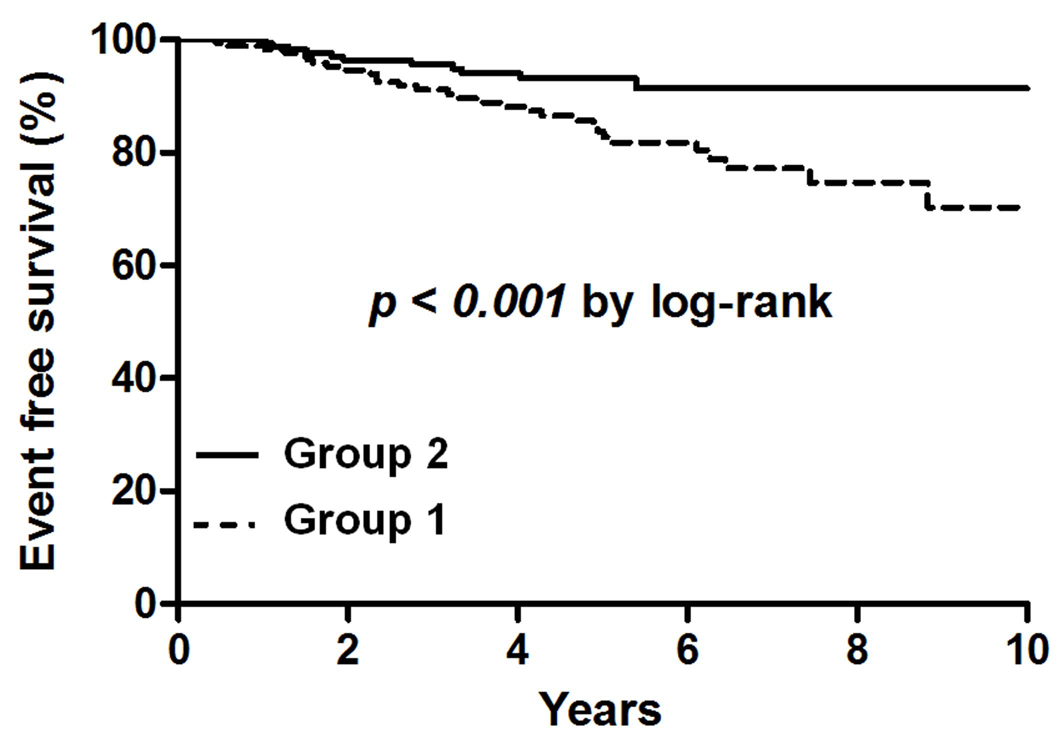

Supplement: S1 Fig — (TIF) [file pone.0166246.s001.tif]
