# Supplementary material for: Statin Intensity and Clinical Outcome in Patients with Stable Coronary Artery Disease and Very Low LDL-Cholesterol
Source: PLoS One. 2016 Nov 8;11(11):e0166246. doi: 10.1371/journal.pone.0166246 (PMC5100958; doi:10.1371/journal.pone.0166246)
Supplement: S1 Table — (DOCX) [file pone.0166246.s003.docx]

**S1 Table. Frequency, doses, and treatment duration of statins in each group.**

| Group 1 (n=181) | | | Group 2 (n=268) | | |
| --- | --- | --- | --- | --- | --- |
| Statins | Frequency (%) | Duration(yrs) | Statins | Frequency (%) | Duration(yrs) |
| Rosuvastatin 5 mg | 11 (6.1) | 4.9 ± 2.7 | Rosuvastatin 20 mg | 34 (12.7) | 3.2 ± 2.2 |
| Atorvastatin 10 mg | 109 (60.2) | 5.2 ± 2.4 | Rosuvastatin 10 mg | 168 (62.7) | 4.5 ± 2.7 |
| Atorvastatin 5 mg | 2 (1.1) | 6.0 ± 0.3 | Atorvastatin 40 mg | 17 (6.3) | 4.1 ± 2.0 |
| Simvastatin 20 mg | 15 (8.3) | 4.5 ± 2.9 | Atorvastatin 20 m | 35 (13.1) | 4.6 ± 2.2 |
| Pitavastatin 2 mg | 30 (16.6) | 4.9 ± 2.8 | Simvastatin 40 mg | 4 (1.5) | 4.6 ± 0.8 |
| Pravastatin 40 mg | 20 (11.0) | 2.6 ± 2.4 | Pitavastatin 4 mg | 4 (1.5) | 3.9 ± 2.9 |
