# Supplementary material for: Statin Intensity and Clinical Outcome in Patients with Stable Coronary Artery Disease and Very Low LDL-Cholesterol
Source: PLoS One. 2016 Nov 8;11(11):e0166246. doi: 10.1371/journal.pone.0166246 (PMC5100958; doi:10.1371/journal.pone.0166246)
Supplement: S2 Table — (DOCX) [file pone.0166246.s004.docx]

**S2 Table. Baseline characteristics of the patients with propensity score matching**

|  | Group 1 (n=181) | Group 2 (n=181) | p |
| --- | --- | --- | --- |
| Age, years | 65±10 | 65±11 | 0.90 |
| Male | 121(66.9) | 130(71.8) | 0.36 |
| Medical history |  |  |  |
| Hypertension | 131(72.4) | 132(72.9) | 1.00 |
| Diabetes mellitus | 66(36.5) | 86(47.5) | 0.04 |
| Current smoker | 38(21.0) | 32(17.7) | 0.51 |
| Body mass index, kg/m^2^ | 24.8±4.3 | 24.6±3.9 | 0.61 |
| Number of diseased vessels |  |  |  |
| 1 | 90(51.4) | 81(44.8) | 0.39 |
| 2 | 50(27.6) | 53(29.3) |  |
| 3 | 38(21.0) | 47(26.0) |  |
| Medications |  |  |  |
| Antiplatelet agents | 181(100) | 181(100) | 1.00 |
| β-blockers | 109 (60.2) | 100 (55.2) | 0.34 |
| Calcium channel blockers | 77 (42.5) | 68 (37.6) | 0.39 |
| ACE inhibitors or ARBs | 89 (49.2) | 91 (50.3) | 0.92 |
| Revascularization |  |  |  |
| PCI | 170 (93.9) | 156 (86.2) | 0.02 |
| CABG | 11(6.1) | 25(13.8) |  |
| Median follow up, years (IQR) | 4.9 (2.3, 6.5) | 4.2 (2.7, 5.8) | 0.11 |

Values are presented as mean ± SD, or n (%) unless otherwise stated; ACE: angiotensin converting enzyme;

ARB: angiotensin receptor blocker; PCI: percutaneous coronary intervention; CABG: coronary artery bypass

graft; IQR: interquartile range
