# Supplementary material for: Statin Intensity and Clinical Outcome in Patients with Stable Coronary Artery Disease and Very Low LDL-Cholesterol
Source: PLoS One. 2016 Nov 8;11(11):e0166246. doi: 10.1371/journal.pone.0166246 (PMC5100958; doi:10.1371/journal.pone.0166246)
Supplement: S3 Table — (DOCX) [file pone.0166246.s005.docx]

**S3 Table. Changes in lipid profiles with propensity score matching**

|  |  | Group 1 (n=181) | Group 2 (n=181) | p^a^ |
| --- | --- | --- | --- | --- |
| Total cholesterol | Before | 151±37 | 156±47 | 0.29 |
|  | After | 129±37 | 120±23 | 0.007 |
|  | p^b^ | <0.001 | <0.001 |  |
| Triglycerides | Before | 117(73, 191) | 97(74, 144) | 0.69 |
|  | After | 112(82, 155) | 102(71, 124) | 0.66 |
|  | p^b^ | 0.002 | <0.001 |  |
| Log Triglycerides | Before | 4.80±0.64 | 4.77±0.57 | 0.35 |
|  | After | 4.62±0.49 | 4.62±0.49 | 0.65 |
|  | p^b^ | <0.001 | <0.001 |  |
| HDL-C | Before | 42.0±10.7 | 43.1±10.9 | 0.38 |
|  | After | 42.0±10.7 | 43.1±10.9 | 0.50 |
|  | p^b^ | 0.001 | 0.44 |  |
| LDL-C | Before | 65±13 | 66±12 | 0.37 |
|  | After | 57±18 | 52±14 | 0.002 |
|  | p^b^ | <0.001 | <0.001 |  |
| % change of LDL-C |  | -7.7±41.8 | -18.1±29.7 | 0.006 |

Values are presented as mean±SD except triglycerides, which is presented as median (IQR); a: comparision

between groups; b: comparison in a group before and after treatment; HDL-C: high-density lipoprotein-

cholesterol; LDL-C: low-density lipoprotein-cholesterol
