# Supplementary material for: Statin Intensity and Clinical Outcome in Patients with Stable Coronary Artery Disease and Very Low LDL-Cholesterol
Source: PLoS One. 2016 Nov 8;11(11):e0166246. doi: 10.1371/journal.pone.0166246 (PMC5100958; doi:10.1371/journal.pone.0166246)
Supplement: S4 Table — (DOCX) [file pone.0166246.s006.docx]

**S4 Table. Incidence of MACE with propensity score matching**

|  | Group 1 (n=181) | Group 2 (n=181) | p |
| --- | --- | --- | --- |
| MACE | 30 (16.6) | 6 (3.3) | <0.001 |
| Cardiovascular death | 3 (1.7) | 1 (0.6) | 0.37 |
| Nonfatal MI | 0 (0) | 1 (0.6) | 1.00 |
| Coronary revascularization | 27 (14.9) | 4 (2.2) | <0.001 |

Values are presented as n (%); MACE: major adverse cardiac events; MI: myocardial infarction
