# Supplementary material for: Statin Intensity and Clinical Outcome in Patients with Stable Coronary Artery Disease and Very Low LDL-Cholesterol
Source: PLoS One. 2016 Nov 8;11(11):e0166246. doi: 10.1371/journal.pone.0166246 (PMC5100958; doi:10.1371/journal.pone.0166246)
Supplement: S5 Table — (DOCX) [file pone.0166246.s007.docx]

**S5 Table. Determinants of MACE according to Cox proportional hazard analysis with propensity**

**score matching**

|  | Unadjusted HR  (95% CI) | p | Adjusted HR  (95% CI) | p |
| --- | --- | --- | --- | --- |
| Age | 1.05 (1.01-1.09) | 0.20 | 1.05 (1.01-1.09) | 0.03 |
| Male | 1.02 (0.49-2.13) | 0.95 | -- | -- |
| Hypertension | 1.1 (0.42-1.84) | 0.73 | -- | -- |
| Diabetes mellitus | 0.96 (0.49-1.87) | 0.90 | -- | -- |
| Current smoker | 1.08 (0.49-2.39) | 0.85 | -- | -- |
| Log Triglycerides | 0.99 (0.55-1.77) | 0.97 | -- | -- |
| HDL-C | 0.96 (0.93-0.99) | 0.02 | 0.96 (0.93-0.99) | 0.04 |
| LDL-C | 0.99 (0.96-1.01) | 0.31 | -- | -- |
| CABG | 1.30 (0.50-3.36) | 0.59 | 1.52 (0.58-3.99) | 0.39 |
| Statin intensity |  |  |  |  |
| Group 1 | Reference | -- | Reference | -- |
| Group 2 | 0.23(0.10-0.56) | 0.001 | 0.22 (0.09-0.54) | 0.001 |

MACE: major adverse cardiac events; HR: hazard ratio; CI: confidence interval; HDL-C: high-density

lipoprotein-cholesterol; LDL-C: low-density lipoprotein-cholesterol; CABG: coronary artery bypass graft
